# Automatically evaluating balance using machine learning and data from a single inertial measurement unit

**DOI:** 10.1186/s12984-021-00894-4

**Published:** 2021-07-13

**Authors:** Fahad Kamran, Kathryn Harrold, Jonathan Zwier, Wendy Carender, Tian Bao, Kathleen H. Sienko, Jenna Wiens

**Affiliations:** 1 Computer Science and Engineering, University of Michigan, Ann Arbor, USA; 2 Mechanical Engineering, University of Michigan, Ann Arbor, USA; 3 Department of Otolaryngology, Michigan Medicine, Ann Arbor, USA

**Keywords:** Balance training, Wearable sensors, Machine learning, Telerehabilitation

## Abstract

**Background:**

Recently, machine learning techniques have been applied to data collected from inertial measurement units to automatically assess balance, but rely on hand-engineered features. We explore the utility of machine learning to automatically extract important features from inertial measurement unit data for balance assessment.

**Findings:**

Ten participants with balance concerns performed multiple balance exercises in a laboratory setting while wearing an inertial measurement unit on their lower back. Physical therapists watched video recordings of participants performing the exercises and rated balance on a 5-point scale. We trained machine learning models using different representations of the unprocessed inertial measurement unit data to estimate physical therapist ratings. On a held-out test set, we compared these learned models to one another, to participants’ self-assessments of balance, and to models trained using hand-engineered features. Utilizing the unprocessed kinematic data from the inertial measurement unit provided significant improvements over both self-assessments and models using hand-engineered features (AUROC of 0.806 vs. 0.768, 0.665).

**Conclusions:**

Unprocessed data from an inertial measurement unit used as input to a machine learning model produced accurate estimates of balance performance. The ability to learn from unprocessed data presents a potentially generalizable approach for assessing balance without the need for labor-intensive feature engineering, while maintaining comparable model performance.

## Introduction

Balance training leverages the ability of the central nervous system to “reweight” functioning sensory inputs to compensate for sensory loss [1]. Balance exercises have been shown to be effective for preventing falls in at-risk individuals with balance concerns, such as older adults and individuals with vestibular deficits [1–4]. But, the most effective programs require the supervision of a physical therapist (PT) [5–7]. Without direct supervision from a PT, remote supervision scenarios (e.g., home-based balance training) provides limited benefits [8, 9]. In such scenarios, PTs typically provide individuals with paper-based instructions along with guidance regarding how often to perform the exercises [10]. Progression through a home-based balance training program is commonly informed by both an individual’s self assessment of their performance in addition to in-person evaluation during clinic-based training sessions [11–13]. However, the lag in real-time feedback associated with this approach could potentially hinder rehabilitation progress and negatively affect rehabilitation outcomes. Moreover, self-assessments may be inaccurate relative to PTs’ assessments, resulting in suboptimal or even unsafe exercise training [14, 15].

To improve the effectiveness of remote rehabilitation training, automated techniques for assessing balance that do not rely on self-assessments or PT supervision are needed. Recently, researchers have investigated the utility of machine learning (ML) tools applied to data collected from inertial measurement units (IMUs) for automatic balance assessment [16]. Such tools have the potential to support quick and accurate estimates of balance performance, thereby improving in-home balance training outcomes and maintaining safe exercising practices. Wearable sensors that measure motion throughout an individual’s daily routine provide a unique opportunity to detect, assess, and evaluate body movement [17, 18]. Like Bao et al. [16], we used data from a single IMU capturing trunk sway data to automatically assess balance. However, in contrast to Bao et al. who relied on an approach that required hand-engineered features, we explored the utility of ML approaches that leveraged unprocessed data. We hypothesized that ML models that leveraged the spatial information during a balance exercise would lead to more accurate assessments of balance compared to other representations. Overall, we focused on the development of methods for providing real-time assessments of balance that were more accurate than self-assessments to provide real-time feedback and improve the effectiveness of remote rehabilitation training methods.

## Methods

### Participants

Balance data were collected from ten participants with self-reported balance concerns. Prospective participants were included if they could stand for 10 minutes without an assistive device and if they did not self report a recent (within the past six months) fall resulting in serious injury or hospitalization, or lower extremity injury (e.g., a lower extremity fracture, sprain) that reduced strength or sensation in their legs. The average age across all recruited participants was 65.7 (± 14.7) years. Of the ten participants, there were four females and six males. Each participant experienced some level of balance concerns, due to either an existing vestibular diagnosis (four participants) or due other circumstances (such as older age). Along with individuals with balance concerns, eight PT participants (subsequently referred to as PTs) with specialization in treating individuals with balance disorders were recruited to assess participant balance. The study protocol was reviewed and approved by the University of Michigan Institutional Review Board. Informed consent was received from all participants and recruited PTs.

### Study design

Each participant performed three 30 s repetitions of 15 standing balance exercises from a group of 54 exercises, while using an overhead harness for safety. The postural exercises were chosen from a standard set used for balance rehabilitation [12]. These exercises varied in difficulty and challenged balance in different (and possibly multiple) ways. Exercises varied in terms of the surface on which they were performed (firm or foam), leg stance (feet apart, feet together, partial heel-to-toe, heel-to-toe, and single-leg), visual condition (eyes open/closed), and head movements (none, pitch head movements, and yaw head movements). Participant balance ability was assessed using a standard set of balance exercises prior to the start of data collection. Based on their baseline abilities, a PT with expertise in balance rehabilitation used a balance exercise progression framework  [12] to select specific exercises for each participant. The cumulative distribution of exercises performed across all participants was considered on a rolling basis when selecting exercises for a given participant to ensure that a broad range of exercises was performed across all participants. We collected a total of 450 exercise repetitions across all participants. Based on video recordings of the exercises, the recruited PTs rated each exercise repetition on a scale from one to five [16]. A label of one represented an exercise that was performed independently with limited or reduced sway, while a five represented an exercise for which the participant was unable to maintain position even with assistance. Among the eight PTs recruited for the study, one to five PTs rated each exercise, with an average of 4.28 (± 0.66) PTs per exercise. We summarized scores by taking the mode among all PT ratings. In addition, participants were asked to rate their own performance using a similar scale [16]. Both scales were purposefully designed to have five points and were adapted from previously published scales [19, 20]. In our analyses, we excluded a small number of exercise repetitions due to missing labels (n = 3) or premature termination of the recording (n = 4).

### Data collection

During the exercises, participants wore a single (six degree of freedom) inertial measurement unit (IMU; MTx, XSens Inc, Eschende, Netherlands) on an elastic belt approximately positioned over the L3 vertebrae level dorsal to the spine to measure trunk sway relative to gravity, in both the pitch and roll directions [21, 22]. For each balance exercise, only angular velocity data were considered as linear acceleration data were not stored. Angular velocity data were sampled at 100 Hz. Although a subset of the exercises involved participants making head movements about the pitch and yaw axes to further challenge participants’ balance for certain stance conditions, only trunk movements about the pitch and roll axes were analyzed from the single trunk-based IMU. These data best capture postural stability in the anterior–posterior and medial–lateral directions and are conventionally reported for kinematic studies using IMUs [16]. We did not apply any preprocessing prior to using the IMU data as input to the model. We also collected ‘step-out’ information, where a step-out indicated any loss of balance resulting in hand contact with the spotter or nearby chair for support, or the need to take a step in order to regain balance. Following a step-out, an individual was encouraged to continue the balance exercise until the full 30 s duration of the repetition had elapsed.

### Machine learning techniques

**Fig. 1:**
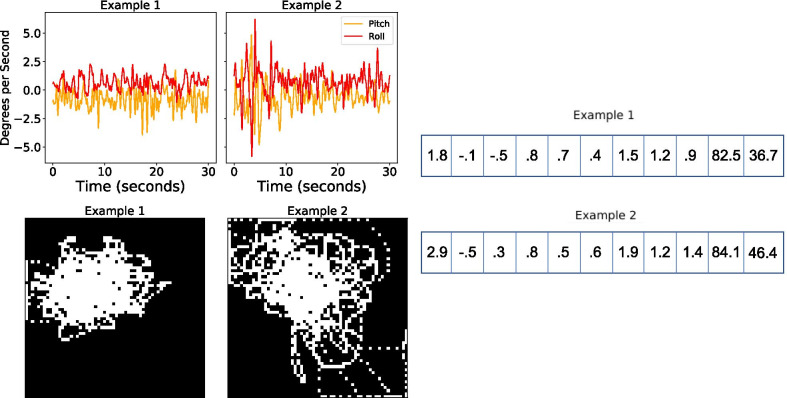
Different representations of the IMU data for two example exercises. Example 1 was rated as a 1, and Example 2 was rated as a 3. The top left shows an example of a time-series representation of the unprocessed IMU data, the bottom left shows an example of an image representation of the IMU data, and the right shows example feature vectors hand-engineered from the IMU data. Example 1 and Example 2 were both taken from the training set. The numbers reported in the feature vector representation represent the statistical descriptors (e.g., root-mean-square of trunk sway in all directions, the path length of the trunk sway trajectory,and the elliptical fit area of the trunk sway ) for these examples used as input to the random forest model

Given these data, we aimed to learn a mapping *f* from a particular representation of the IMU data $x \in {\mathcal {X}}$ to a summarized PT label $y \in {\mathcal {Y}} = \{1,2,3,4,5\}$ (based on the mode). To learn potentially complex non-linear relationships between the IMU data and the summarized PT label, we considered using the IMU data as input to different machine learning models. Each model was trained to accurately estimate the PT label on a set of training data, before being applied to a held-out set of test data to assess generalization. However, there are many different techniques for representing IMU data for a particular exercise repetition. We considered three different representations of the IMU data as inputs to a machine learning model (Fig. 1). Based on the input, each model produced an estimate of balance performance in $\{1,2,3,4,5\}$, with the goal of matching the PT labels. First, we considered a multivariate time-series representation of the data with two channels (for pitch and roll, respectively). A time-series representation encoded the temporal dependencies present in the data. We used the time-series data as input to a 1-dimensional convolutional neural network (CNN), which has previously been shown to be an effective model for learning from time-series data [23–25]. Second, we considered an image representation of the IMU data. To create an image representation, we plotted pitch on the x-axis and roll on the y-axis. We then transformed this plot into a 2-D $60 \times 60$ image, that could be used as input to a model. This image was used as the input to a 2-dimensional CNN. Among these two representations of the IMU data, we hypothesized that the image representation would outperform the time-series representation, as the ability to assess balance likely relies on the raw spatial pitch and roll information more than the temporal relationship of pitch and roll. Third, we also represented each exercise by extracting 11 features that had previously been shown to be useful in assessing balance [16, 26–28]. In particular, we calculated the kinematic features that were used by Bao et al. for each repetition: the root-mean-square of trunk sway in all directions, the path length of the trunk sway trajectory, and the elliptical fit area of the trunk sway [16]. We used this feature vector as input to a random forest model.

### Experimental set-up

To train and evaluate our models, we split our data into a training dataset, a validation dataset, and a testing dataset based on the participant. Specifically, we used data from participants 1–6 as the training set, data from participants 7 and 8 as our validation set, and data from participants 9 and 10 as our test set. Given the computational costs associated with training ML models, we considered only a single held-out test set, as in past work [29, 30]. However, the distribution of summarized PT labels was consistent across our training, validation, and test sets.

Models were optimized by minimizing the cross-entropy loss. We used the Adam optimizer with a learning rate of $1 \times 10^{-4}$, a batch size of 32 and weight decay tuned for each specific model [31, 32]. Each model was trained with a fixed budget of 2000 epochs, and hyperparameters (such as weight decay) were chosen with respect to the performance on the validation set. When training the 2-dimensional CNN using the image representation, we augmented our dataset by randomly rotating each image in the training set three separate times, with each rotation randomly taken from the set $\{30^\circ , 60^\circ , 120^\circ , 150^\circ , 210^\circ , 240^\circ , 300^\circ , 330^\circ \}$. We selected the hyperparameters of the random forest, specifically the number of trees (1000), by using grid search and a leave-one-participant-out cross validation scheme to maximize the performance on the held-out set.

For the 1-dimensional CNN, we used one convolutional layer with eight filters and a kernel size of three, followed by max pooling, ReLU activation, and batch normalization. We experimented with more convolutional layers, yet saw no improvement in performance. We followed this convolutional block with two fully-connected layers, with batch normalization and ReLU activation in between [33, 34]. To reduce over-fitting, we applied dropout with probability 0.5 following the first fully-connected layer [35]. For the 2-dimensional CNN, we used eight filters and a kernel size of 3 × 3, followed by a similar architecture as used in the 1-dimensional CNN. Given that position and velocity likely encode different information, we used depth-wise convolutions resulting in different filters for each input type for both CNN architectures [36].

We evaluated models in terms of classification accuracy and the area under the receiver operating characteristics curve (AUROC). Accuracy indicated how often our model aligned with how a PT might have assessed performance during an exercise. The AUROC is a common evaluation metric that measures a model’s capability of correctly ranking examples. Given our multiclass setting, we considered the macro-averaged AUROC, which computed the average value among the AUROC calculated across each class independently. We used validation AUROC as the performance metric during hyperparameter selection. For additional context, we compared the discriminative performance of the ML methods to two additional baselines: (1) naively predicting the mode label (i.e., ‘2’) for all ratings in the test set (which we term the ‘majority classifier’) and (2) using a participant’s self-assessment ratings as predictions for each exercise. Comparing to the simple majority classifier tested whether or not the ML models were learning something beyond the mode, while comparing to the self-assessment baseline demonstrated the ability of the ML models to improve upon an individual’s self-assessment when estimating ground-truth PT assessments of balance. All experiments were repeated 30 times with different random initializations of the model to evaluate stability. Throughout the rest of the paper, we report the average accuracy and AUROC over the 30 runs on the test set, as well as the standard deviation (SD). We implemented all neural network-based approaches in PyTorch. We trained each model on a GeForce GTX 1080 Ti GPU.

### Statistical analysis

We repeatedly trained models based on 30 different random initializations of the model parameters. To compare the performance of our models, we used paired *t*-tests to test for significant differences of the mean performance of each method across 30 runs with different random initializations. A paired *t*-test was used as performance was calculated for each model on the same examples in the test set, making the examples related. Significant differences were defined at a significance level of $\alpha = 0.05$.

## Results

On average, each exercise was rated by four PTs (out of a total of eight PTs), and a random PT’s rating agreed with the mode rating for an exercise repetition 77.7% of the time throughout the whole dataset, and 71.1% in the test set. In addition, we considered the AUROC of a random PT compared to the mode label. Specifically, we randomly selected one PT’s label as the predicted label for that exercise, and calculated the AUROC of those predictions relative to the mode label. Repeating this procedure 1000 times, the ‘average PT’ achieved an average AUROC of 0.859 (± 0.012) on the entire dataset, and an average AUROC of 0.805 (± 0.033) on the test dataset. These results suggest an expected upper-bound on model performance. The AUROC is typically upper-bounded by 1, but given the task difficulty we did not expect to perform much better than a randomly selected PT, as the target labels were based on PT assessments. Moreover, the Krippendorf’s alpha for ratings was 0.013, representing low agreement among PTs [37].

**Fig. 2:**
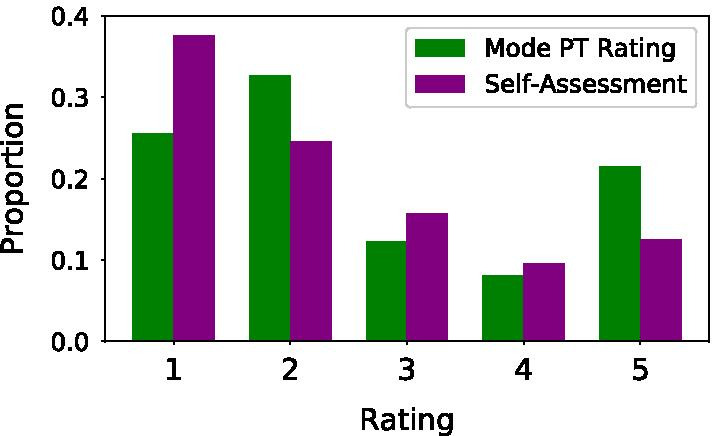
The distribution of PT ratings vs. self-assessment ratings. A majority of the exercises were labelled as either a 1, 2, or a 5. In general, the self-assessment ratings tended to underestimate the PT ratings

Self-assessment ratings tended to be over confident compared to the ground-truth PT ratings, with an average self-assessment of 2.35 (± 1.39) and substantially more 1 ratings, compared to an average PT rating of 2.67 (± 1.48) with substantially more 5 ratings (Fig. 2). Many examples of a ground-truth label of 5 were assessed by a participant as a label of 3 or 4, and many examples of a ground-truth label of 2 were self-assessed as a 1. ML models significantly outperformed the majority classifier and the self-assessments in terms of both accuracy and AUROC (Table 1). In terms of AUROC, both models directly leveraging the IMU data significantly outperformed the models trained using the hand-engineered features in terms of AUROC ($$p < 0.05$$). In terms of the accuracy, there was no significant difference between the best performing model (which was based on hand-engineered features) and the next best (based on an image representation) ($$p > 0.05)$$.

**Table 1:** Comparing different ML architectures and representations

Representation	Test accuracy (%)	Test AUROC
Majority classifier	$37.8\% \pm 0.0\%$	$0.500 \pm 0.000$
Self-assessments	$43.3\% \pm 0.0\%$	$0.665 \pm 0.000$
Engineered features	$\mathbf {57.2\% \pm 1.0\%}$	$0.768 \pm 0.007$
Time-series	$55.1\% \pm 2.2\%$	$0.801 \pm 0.004$
Image	$56.4\% \pm 1.3\%$	$\mathbf {0.806 \pm 0.002}$

All results show a clear improvement over both non ML baselines. The time-series representation as input to a 1D CNN model outperformed the engineered features in terms of AUROC, while the 2D CNN using an image representation as input resulted in the best performance. Bold signifies the best performing model for accuracy and AUROC respectively

Across all models, the models performed best at predicting labels of 2 and 5 (accuracy of 61% for a label of 2 and 85% for a label of 5), followed by a label of 1. Compared to predicting other labels, models performed poorly when estimating labels of 3 and 4. We posit that this outcome was due to the low prevalence of these labels in the dataset. Moreover, examples with a label of 3 were often classified across all ML models as a label of 2, pointing to a potentially difficult distinction between 2s and 3s that the model was unable to detect.

## Discussion

Overall, we demonstrated that ML models can accurately assess balance based on unprocessed kinematic data collected from an IMU, outperforming recently proposed feature-based approaches [16]. Even with limited training data, models using either time-series or image-based representations as input achieved significantly better AUROC compared to a model trained using engineered features as input. Circumventing the need for extracting hand-engineered features (that may fail to generalize across different tasks) eliminates a labor-intensive step in an ML pipeline. These results demonstrate the potential of using unprocessed IMU data as input to ML models for building automatic balance assessment methods.

At-home balance training with remote supervision allows PTs to progress individuals through exercises based on individual self-assessments. However, self-assessments may be inaccurate relative to a PT's assessment, resulting in suboptimal or even unsafe exercise training [14, 15]. Compared to self-assessments, we showed that ML models, and in particular those that leveraged the unprocessed IMU data, resulted in consistently better AUROC, with the best ML model achieving an AUROC of 0.806 (vs. 0.665 for the self-assessments). Moreover, though accuracy was well below 100%, the performance of the ML algorithms represent a significant improvement over self-assessments (56.4% vs. 43.3%). Thus, replacing participant self-assessments with automatically-generated model assessments could lead to potential improvements in at-home balance rehabilitation training. 

The ML models improved upon an important weakness of self-assessments by correctly classifying repetitions that were too challenging (i.e., labelled as 5). In contrast, self-assessments tended to be over confident when labeling challenging exercise repetitions. The more conservative assessments produced by the ML model in such scenarios may reduce the likelihood of an individual performing balance exercises that are too challenging. This in turn could improve the safety of at-home balance rehabilitation programs.

Comparing the ML models that used unprocessed data, the image-based model outperformed the time-series model. This result suggests that spatial information associated with pitch and roll signals may be more important than temporal dependencies when assessing balance. Moreover, a model leveraging an image-based representation can apply to balance exercises of different lengths. Hence, an image representation of the angular velocity IMU data as input to a model presents a flexible and accurate method for building models for real-time balance assessment going forward.

We note two major limitations of our work. First, as multiple different sets of PTs rated each exercise separately, there was no consensus PT rating on which to train our models. To overcome this issue, we took the mode label, a natural choice for summarizing multiple votes. However, future work could consider more robust scales and PT rating labels to ensure noise-free target labels in the case of disagreement among multiple PTs for a particular exercise repetition. Despite this limitation, the strong results of the ML models when training using the mode PT label are encouraging and they show the potential of learning from unprocessed IMU data even when noise exists in the target label. Second, our dataset was relatively small and consisted of a limited subset of balance training exercises. These limitations precluded us from exploring more complex state-of-the-art ML architectures. However, the ability of simple ML architectures to achieve strong performance with limited training data is promising. As we amass more data from wearable sensors and build more balance exercise datasets, the findings from this work can help inform ML architectures that can further improve performance on the task of balance assessment. Overall, these results demonstrate the potential for ML models utilizing unprocessed IMU data to improve at-home balance rehabilitation training schemes by supporting the delivery of accurate and timely feedback.

## Data Availability

The data collected and/or analyzed in this study are available from the corresponding author on reasonable request.

## Abbreviations

IMU: Inertial measurement unit
PT: Physical therapist
AUROC: Area under the receiver operating characteristic curve
NN: Neural network
RNN: Recurrent neural network
LSTM: Long short-term network
CNN: Convolutional neural network
